# Deep Sequencing Reveals the Complete Genome and Evidence for Transcriptional Activity of the First Virus-Like Sequences Identified in *Aristotelia chilensis* (Maqui Berry)

**DOI:** 10.3390/v7041685

**Published:** 2015-04-03

**Authors:** Javier Villacreses, Marcelo Rojas-Herrera, Carolina Sánchez, Nicole Hewstone, Soledad F. Undurraga, Juan F. Alzate, Patricio Manque, Vinicius Maracaja-Coutinho, Victor Polanco

**Affiliations:** 1 Centro de Genómica y Bioinformática, Facultad de Ciencias, Universidad Mayor, Santiago 8580000, Chile; E-Mails: javier.villacreses@mayor.cl (J.V.); marcelo.rojas@umayor.cl (M.R.-H.); carlina.sanchezd@umayor.cl (C.S.); soledad.undurraga@umayor.cl (S.F.U.); patricio.manque@umayor.cl (P.M.); 2 Centro Nacional de Secuenciación Genómica, Universidad de Antioquia, Medellín, Colombia; E-Mail: jfernando.alzate@udea.edu.co; 3 Maqui New Life, Santiago, Chile; E-Mail: info@mnl-group.com

**Keywords:** *Aristotelia chilensis*, Maqui berry, genome, transcriptome, comparative genomics, deep sequencing, RNA sequencing, *Petuvirus*, *Caulimoviridae*, plant virus

## Abstract

Here, we report the genome sequence and evidence for transcriptional activity of a virus-like element in the native Chilean berry tree *Aristotelia chilensis*. We propose to name the endogenous sequence as *Aristotelia chilensis Virus 1* (AcV1). High-throughput sequencing of the genome of this tree uncovered an endogenous viral element, with a size of 7122 bp, corresponding to the complete genome of AcV1. Its sequence contains three open reading frames (ORFs): ORFs 1 and 2 shares 66%–73% amino acid similarity with members of the *Caulimoviridae* virus family, especially the *Petunia vein clearing virus* (PVCV), *Petuvirus* genus. ORF1 encodes a movement protein (MP); ORF2 a Reverse Transcriptase (RT) and a Ribonuclease H (RNase H) domain; and ORF3 showed no amino acid sequence similarity with any other known virus proteins. Analogous to other known endogenous pararetrovirus sequences (EPRVs), AcV1 is integrated in the genome of Maqui Berry and showed low viral transcriptional activity, which was detected by deep sequencing technology (DNA and RNA-seq). Phylogenetic analysis of AcV1 and other pararetroviruses revealed a closer resemblance with *Petuvirus*. Overall, our data suggests that AcV1 could be a new member of *Caulimoviridae* family, genus *Petuvirus*, and the first evidence of this kind of virus in a fruit plant.

## 1. Introduction

Similar to all eukaryotic organisms, plant genomes commonly have inserted mobile genetic elements and viruses [1]. New high throughput sequencing technologies (DNA and RNA-seq) and bioinformatics methods have accelerated the sequencing and analysis of new genomes and transcriptomes of complex organisms, allowing the identification of new virus species integrated within complete or draft genome sequences [2,3].

Maqui (*Aristotelia chilensis* [Molina] Stuntz) is an endemic Chilean tree. It has a high economic value due to the characteristics of its fruits, which possess a high antioxidant capacity [4]. This fruit’s ability to scavenge reactive oxygen species (ROS) is conferred by its extremely elevated anthocyanin content, which surpasses the levels of other popular berries, *i.e.*, strawberries, blueberries and grapes [4,5]. Moreover, it has been reported that Maqui Berry has anti-inflammatory properties [6], cardioprotective activity [7], inhibits LDL oxidation *in vitro* and protects human endothelial cells against oxidative stress [8]. To date, there is not genetic or phenotypic evidence of viral species in this kind of plants. The goal of this study was to demonstrate the presence of a virus in Maqui, using genomics and bioinformatics tools.

We used next generation sequencing (NGS) to obtain the first draft genome sequence of Maqui berry. This initiative allowed us to uncover for the first time the presence of a new virus inserted in the Maqui genome, named here as: *Aristotelia chilensis Virus 1* (AcV1). Comparative genomics revealed a strong phylogenetic relationship of AcV1 with the *Caulimoviridae* family, genus *Petuvirus*. Viruses from this genus commonly infect plants and replicate by reverse transcription (RT) [9]. The unique member of this genus is the *Petunia vein clearing virus* (PVCV), which is able to integrate within the host’s Petunia genome, and is classified as an endogenous pararetrovirus (EPRV). It affects plants, causing a variety of different phenotypes, like chlorotic tissues, leaf malformation, vein-clearing symptoms and other affections [10]. Besides the strong sequence similarities and phylogenetic relationship between AcV1 and PVCV, these symptoms were not observed in Maqui plants.

Finally, we decided to perform RNA-seq assays in order to obtain the overall transcriptional activity of the virus in different plant tissues. Our results showed that AcV1 genes have a low transcriptional activity, which might be associated with the absence of an infective phenotype. This study constitutes the first report evidencing the presence of a virus in Maqui berry and an important step towards the molecular characterization of mobile genetic elements in this native Chilean tree.

## 2. Results

### 2.1. Deep Sequencing of Aristotelia Chilensis Genome Results in the Discovery of AcV1

Next generation sequencing approaches were used to study the genome of the native Chilean fruit tree *A. chilensis* (sample M152). Data analysis of assembled contigs allowed us to identify the initial signs of viral sequences integrated within the genome. Based on sequence similarities searches using BLASTn against the NCBI nucleotide NT database, we identified a contig sequence with high similarity with viral sequences. This contig contains a length of 12,109 nucleotides (nt), a GC content of 38.04% and is composed of 12,032 reads. This initial similarity analysis showed top 10 BLASTn hits with complete pararetrovirus and EPRV sequences (*e-values* ≤ 2e-05), suggesting the presence of a complete virus inserted in Maqui genome, named here as *Aristotelia chilensis Virus 1* (AcV1), containing a full size estimated in 7122 bp. In order to check if AcV1 was present in a single copy in Maqui berry, we mapped the complete viral nucleotide sequence against all contigs and scaffolds from Maqui genome. It confirmed that the virus was present once in the genome (query coverage ≥ 50%).

### 2.2. Bioinformatics Characterization of AcV1

Open reading frame (ORF) predictions using the software Artemis [11] and BLASTx similarity searches against the NR protein database of NCBI, revealed the presence of three different ORFs along the contig sequence (Table 1). ORF1, the longest, starts on the +3 reading frame of the sense strand of the virus genome sequence and its predicted gene product is composed of 820 amino acids (aa), with a predicted molecular weight (MW) of 92.22 kDa. ORF2 is located at the +2 reading frame of the sense strand and is composed of 758 aa, with a predicted MW of 88.69 kDa. ORF3, the smallest one, is located at the −1 frame of the antisense strand, and is composed of 266 aa, with a predicted MW of 29.91 kDa. The stop codon of ORF1 (TGA, genomic coordinates: 2541–2543) overlaps with the start codon of ORF2 (ATG, genomic coordinates: 2540–2542). Similar characteristics have been previously described in pararetroviruses, such as *Caulimoviruses* [12], *Badnaviruses* [13] and *Tungrovirus* [14].

**Table 1:** AcV1 Open Reading Frames and related gene products. All coordinates are related to the viral sequence within the contig.

ORF Number	Start Position	End Position	Length (bp)	Predicted Length (aa)	Protein Weight (kDa)
1	81	2543	2463	820	92.22
2	2540	4816	2277	758	88.69
3	6208	5408	800	266	29.91

In order to predict the putative functions of the virus ORFs, we used their translated amino acid sequences to perform BLASTp searches. This analysis indicated that ORF1 encodes a Movement protein, whereas ORF2 corresponds to a Reverse transcriptase (RT). On the contrary, ORF3 showed no amino acid sequence similarity with any known virus protein sequence available in the NCBI database (unknown protein). Besides its absence in public databases, together with ORF1 and ORF2, the ORF3 is part of the virus due to its localization between key features available in viral elements: the tRNA binding site in one side, and the poly(A) signal on the other one. Therefore, we conclude that ORF3 is potentially exclusive of AcV1.

**Figure 1:**
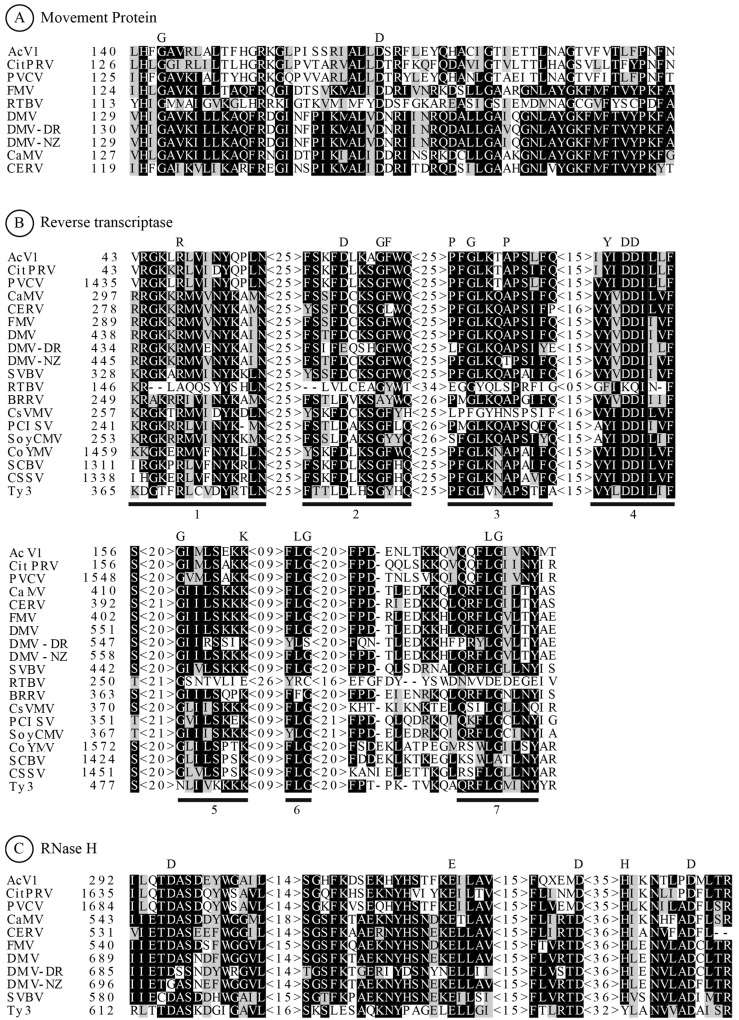
Multiple sequence alignment of conserved domains within AcV1 proteins against selected pararetoviruses and LTR retrotransposon Ty3 from *Saccharomyces cerevisiae*. Pararetroviruses annotated sequences with significant similarity to AcV1 were selected for a multiple sequence alignment. Conserved characteristic domains between AcV1, *Caulimoviridae* family virus and Retrotransposon are highlighted. (**A**) Putative domains of AcV1 ORF1 (MP) were aligned with caulimoviruses previously selected by homology searches: CitPRV, PVCV, FMV, *Rice tungro bacilliform virus* (RTBV) [25], *Dahlia mosaic virus* (DMV), DMV-10-NZ, DMV-10-DR [26], *Cauliflower mosaic virus* CaMV, and CERV; (**B**) AcV1 ORF2 (RT) were aligned with reverse transcriptases (RT) from Caulimoviruses and a selected retrotransposon. Invariant motifs distinctive of RT’s were underlined and numbered as described by Xiong and Eickbush [22]; (**C**) AcV1 ORF2 were aligned with Ribonuclease H regions from different Caulimoviruses and Ty3 retrotransposon [23]. Numbers located next to the retroviruses names indicate the amino acid position within the designated ORF. Conserved amino acid residues within a group are highlighted with a black box. Different amino acids containing similar chemical properties are shown in a gray box. Invariant amino acids are highlighted in boldface above the sequences. G = Gly, D = Asp, R = Arg, Y = Tyr, F = Phe, E = Glu, P = Pro, K = Lys, L = Leu, H = His.

Our alignment searches revealed that ORF1 has high similarity with the movement proteins of PVCV (*e-value* = 8e-106) [15], followed by *Citrus endogenous pararetrovirus* (CitPRV) (*e-value* = 4e-94) [16] and *Figwort mosaic virus* (FMV) (*e-value* = 5e-06) [17]. A deeper exploration using multiple sequence alignments with CLUSTAL OMEGA [18] showed that ORF1 displays a movement protein family I domain, which is conserved in all *Caulimoviridae* family’s movement proteins (plant pararetrovirus) [19]. ORF1 alignments also revealed a 66%, 63% and 20% identity with the movement proteins of PVCV, CitPRV *and Carnation etched ring virus* (CERV) respectively [12] (Figure 1A). Additionally, we found two highly conserved residues Gly (G) and Asp (D), characteristic of MPs, constituting the representative evolution site of the MP protein in *Caulimoviridae* family and several other virus groups [19]. These proteins modify plasmodesmata by forming intra-channel tubules that can accommodate viral particles, comprising one of the most important transport mechanisms used by several plant viruses [20].

The same approach was applied to ORF2, in which BLASTp alignments showed higher scores with RT proteins from PVCV (*e-value* = 1e-173) [15], CitPRV (*e-value* = 1e-149) [16], CaMV (*e-value* = 2e-90) [21], CERV (*e-value* = 8e-83) [12] and FMV (*e-value* = 2e-86) [17]. Multiple alignments showed a 73%, 56%, 38% and 37% identity with PVCV, CitPRV, CaMV and FMV, respectively. The alignments of ORF2 with the reverse transcriptase of different members of *Caulimoviridae* pararetrovirus family and selected retransposon are shown in Figure 1B. Conserved amino acid sequences were identified, representing seven motifs with invariant amino acids: Arg (R) and Tyr (Y) in motif 1; Asp, Gly, and Phe (F) in motif 2; Pro (P), Gly, and Pro in motif 3; Tyr, Asp, and Asp in motif 4; Gly and Lys (K) in motif 5; Leu (L) and Gly in motifs 6 and 7. These motifs are known to be related to the origins and evolution of retrotransposable elements, and are known to be exclusively conserved in RT sequences [15,22]. RT is a key element in retrotransposons, retroviruses, group II introns, bacterial multi-copy single-stranded DNA (msDNA), hepadnaviruses, and Caulimoviruses. RT catalyzes DNA replication from an RNA template, and is responsible for the replication of retroelements [21].

In addition, ORF2 showed a conserved RNase H domain according to BLASTp analysis. Multiple sequence alignments of this domain showed a 64% identity with RNase H protein from PVCV and 27% from Ty3 retrotransposon [23]. RNase H is an endonuclease that cleaves the RNA strand of RNA/DNA hybrid in a sequence nonspecific manner. This characteristic is widely present in various organisms and it is clearly important for its relation with the origins of viruses [21]. The conserved amino acids Asp, Glu, Asp, His (H) and Asp are known to be related to the catalytic region of the protein (Figure 1C). Additionally, we showed that a histidine (His) motif was highly conserved with the pararetrovirus and not conserved with the retrotransposon. This may suggest that the His motif from Rnase H potentially has key roles in viral sequences [24].

### 2.3. Phylogenetic Analysis Reveals a Strong Relationship between AcV1 and the Caulimoviridae Family

**Figure 2:**
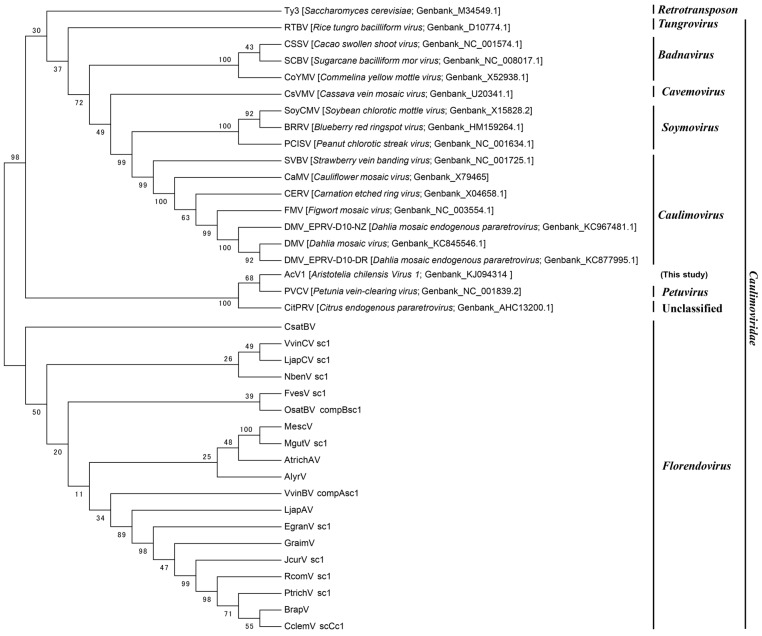
Phylogenetic analysis of retrotranscriptase domains from *Caulimoviridae* family, endogenous pararetrovirus and Ty3 retrotransposon.

Phylogenetic analysis is a powerful tool commonly used to make evolutionary comparisons in order to identify relationships between sequences and different species. Aiming to classify the viral family that AcV1 belongs, we built a phylogenetic tree based on the RT protein sequence from AcV1 in comparison with different members of two endogenous Dahlia viruses [26], CitPRV [16], one representative member of *Florendoviruses* based on the Family of different host plants [27] and Ty3 retrotransposon of *S. cerevisiae* (Figure 2). 

Our results showed that AcV1 has a high phylogenetic relatedness with *Caulimoviridae* family, especially with *Petuvirus*. It gives an additional support that AcV1 is a viral sequence, represented as an endogenous viral sequence. In addition, this analysis revealed that each genus of the *Caulimoviridae* family clustered separated in different branches. AcV1 clustered together with PVCV and CitPRV, suggesting that they can be part of the same genus: *Petuvirus* (Figure 2). However, it is necessary to have access to a bigger number of related viruses sequences and experimental assays in order to validate this hypothesis.

Also, in order to verify if each one of the viruses represented on the phylogenetic tree was also inserted in Maqui berry, we compared their complete sequence against all contigs and scaffolds from the Maqui genome. It showed that only AcV1 was present in Maqui. However, when we compared only the nucleotide sequences of AcV1 ORF2, containing both RT and RNase H domains, against Maqui genome, we found 66 copies using a query coverage bigger than 80% and 87 copies with a coverage above 60%. It might represent rearrangements of AcV1 or the RT and RNase H domain sequences of other still uncharacterized endogenous pararetroviral sequences available in the Maqui genome, that conserved the RT and RNase H domains.

Phylogenetic analysis was implemented using Maximum Likelihood [28]. The evolutionary distances were computed using the Dayhoff matrix-based approach [29]. The bootstrap consensus tree representing the evolutionary history of the taxa analyzed was inferred from 1000 replicates. Nodes below the bootstrap threshold were collapsed [30]. Evolutionary analyses were conducted using MEGA 5 [28]. Genbank accession numbers for each *Caulimoviridae* family virus and Ty3 retroelement are shown. *Florendovirus* sequences are still not available on GenBank, their sequences were extracted from Geering *et al.* [27], and are available on the Supplementary File 1.

### 2.4. General Genomic Organization of AcV1

**Figure 3:**
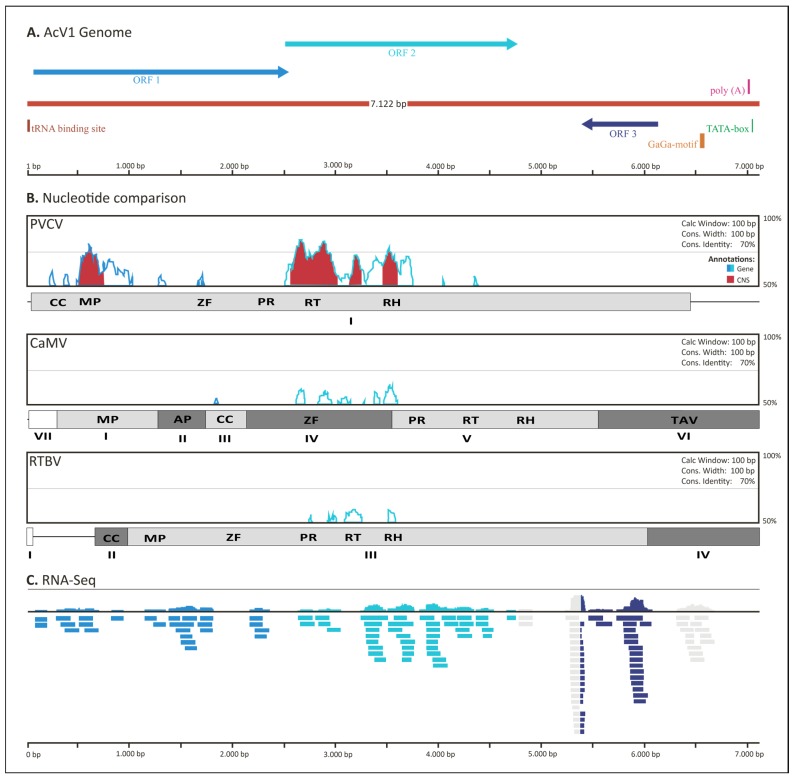
Overview of AcV1 genomic sequence, its transcription in Maqui berry and conservation with related virus. (**A**) AcV1 gene annotations and genomic features: ORF1, movement protein (blue); ORF2, reverse transcriptase and Rnase H domain (light blue); ORF3, unknown protein (dark blue); tRNA binding site (brown); GaGa motif (orange); poly(A) (pink); TATA-box (green); (**B**) Conservational whole genomic comparisons between AcV1 and different related viruses: PVCV, CaMV and RTBV. Genes have its coding regions (ORFs) represented in light blue and light green lines, and conserved non-coding sequences (CNS) are filled with red. The ORFs and protein domains are represented as: MP, Movement Protein; ZF, zinc finger; PR, protease; RT, reverse transcriptase; RH, RNAse H; AP, aphid transmission; TAV, transactivator/viroplasmin; (**C**) *A. chilensis* transcriptome reads distribution along AcV1 genome. The color is represented according to the overlapping of genomics coordinates between RNA reads and the predicted ORFs.

Using a bioinformatics approach we identified that AcV1 is a single-copy endogenous pararetrovirus inserted in Maqui berry genome. The full length of AcV1 was estimated in 7122 bp, similar to other pararetroviruses from the *Caulimoviridae* family (Figure 3A). We found that AcV1 is smaller in 87 bp than PVCV (length: 7206 bp) [15]. Also, it is 1036 bp smaller than CsVMV virus (length: 8159 bp) [31]. Amongst plant retroviruses, the smallest viruses reported are the *Badnavirus Cacao swollen shoot virus* (CSSV), 7161 bp long [13] and the *Petuvirus* PVCV, 7205 bp long. Based on previous reports, AcV1 could be classified as a small plant virus [13,15,31].

Furthermore, we found a short non-coding sequence, located 80 bp upstream of the ORF1, that has complementarity with the first 14 nucleotides of the 3' strand methionine tRNA, which was previously described in plants (TGGTATCAGAGCCA, AcV1 genomic coordinates: 1–14, Figure 3A) [12,32]. This region is predicted to be the first nucleotides of the priming binding site for replication by reverse transcription, representing the beginning of AcV1 sequence in the genome of *A. chilensis*. 

In addition, we found a TATA box region (TATATAA, AcV1 genomic coordinates: 7038–7044) and a GAGA motif (AGAGAGAGAGAGAGAG, AcV1 genomic coordinates: 6697–6712) located in the antisense strand, downstream to ORF3. This promoter structure is similar to the downstream promoter sequence (dps) of RTBV, which is known to contribute with its transcriptional activity. The efficacy of this promoter in RTBV is associated with the genomic localization of the GAGA motif, which might be located immediately downstream to the TATA-box [33]. Also, we found a poly(A) signal (AATAAAA, AcV1 genomic coordinates: 7025–7031) located downstream of ORF3.

The genomic coordinates of these motifs and the localization of each predicted gene were used in the estimation of the whole viral genome length and its organization. A general whole genome conservational analysis between AcV1 and three related virus from the *Caulimoviridae* family (PVCV, CaMV and RTBV) was performed (Figure 3B). Alignments using MLAGAN [34], through VISTA tool [35] and displayed as “peaks and valleys” graphics showed a concordantly genomic structure, especially with PVCV. The conserved region between coordinates 1 and 2000 bp showed to be specific of the *Petuvirus* genus, especially due to the similar structural organization and the presence of the MP sequence. The genomic coordinates between 2500 and 4000 bp is conserved between all pararetroviruses, it is the region that hosts the RT and RNAse H domains. Finally, the region between 5000 and 7122 bp is not conserved. Interestingly, this region hosts the predicted ORF3, which was shown in our similarity analysis to be exclusive of AcV1.

### 2.5. Evidence of AcV1 Transcriptional Activity Based on Expression Levels Estimated by RNA-seq

*A. chilensis* has become an important case of study for plant research in Chile. Numerous studies reporting different insights of its biological properties and high antioxidant capacity have been published [6,7,8]. However, by now, there are no reports demonstrating phenotypic alterations in *A. chilensis* caused by viral activity or infection. Therefore, in order to find the viral expression in Maqui plants and obtain a first insight of its activity, we performed Illumina RNA-seq assays in two different specific tissues: fruits under three different developmental stages (green, half-ripe and ripe) and leaf.

A total of 43 million high quality reads from all libraries were mapped against the virus genomic region. Of these, 21 mapped to ORF1 (movement protein), 23 to ORF2 (reverse transcriptase), 11 to ORF3 (unknown protein) (Figure 3C). As observed, the RNA-seq assays in all four samples revealed similar expression levels based on reads count. The results indicated that all tissues and samples had similarly low expression levels, concordantly to the absence of symptoms and the optimal growing conditions of these plant samples. Since this is the first virus described in *A. chilensis*, the absence of symptoms described to date makes it difficult to identify its real effects in the plant.

## 3. Discussion

We describe here the first virus inserted in the genome of the Chilean plant Maqui berry, AcV1, using deep sequencing and bioinformatics approaches. We provided evidence of its phylogenetic relationships, genomic structure and the first support of a potential expression activity. Our data, together with the known characteristics of PRV and EPRV viruses, are important evidence that support the classification of *A. chilensis Virus 1* as an endogenous pararetrovirus, part of the *Caulimovirus* family, and specially related with PVCV from *Petunia hybrid* [15] and CitPRV from *Citrus citrange* [16]. This relationship can suggest that these three viruses (AcV1, CitPRV and PVCV) are part of the same genus: *Petuvirus*. However, additional genome sequences of other viruses and further experimental assays are necessary in order to support this hypothesis.

AcV1 sequences were identified inserted in the Maqui berry genome, suggesting an integration event, available in an episomal single copy identified in the contig sequence. These integration events were previously described for endogenous pararetroviruses, such as *Banana Strike Virus* (eBSV) [36], *Tobacco Vein Clearing Virus* (eTVCV) [37] and the endogenous *Petunia vein-clearing virus* (ePVCV), among others [38,39,40]. Based on comparative genomics approaches, we found a strong relationship between AcV1 and pararetrovirus, especially with the genome of PVCV. This relationship is higher mainly on the regions hosting the main proteins (MP, RT and the RNase H domains), commonly founded in all plant viruses. The main differences are on its genome structure organization, in which some domains, like the Protease (PR) and the Zinc finger motif, are present in PVCV and absent in AcV1; and the region containing the ORF3 in AcV1, which is possible exclusive of this virus. It could be part of integration/deletion events during the endogenous pararetrovirus evolution, as previously observed in different EPRVs [41,42], that are enriched in the *Caulimoviridae* family, and its presence is also strongly related to plants evolution [24,39]. Based on these similarities and differences, AcV1 could also be called eAcV1 (*endogenous Aristothelia chilensis Virus 1*) and classified as a *Petuvirus*. However, Southern blotting assays and transmission experiments using isolated full-length AcV1 clones could give a definitive support to classify AcV1 as a pararetrovirus.

The observed AcV1 transcriptional activity (low expression) is an additional signal of the viral presence and activity. The evidenced small transcriptional activity can be due to the fact that the sequenced Maqui plant was asymptomatic under normal growth conditions, and should be verified by PCR/qPCR or Northern blot assays. The low expression level of viral transcripts is probably associated with the absence of symptomatic phenotype*.* This small expression in EPRV viruses has been shown associated with an epigenetic control regulated by DNA methylation of integrated EPRV genome regions in *Solanaceae* healthy plants [39]. Its de-methylation results in phenotypic alterations associated to viral expression. Studies with ePVCV showed a relationship between DNA methylation and the production of small interfering RNAs. The authors of the research proposed that viral symptoms might be associated to this a de-methylation stage that increases viral transcripts and siRNA expression levels [39]. Bioinformatics exploration of ESTs databases and experimental validation by RT-PCR of various EPRVs in different plant species revealed that its low-level transcription is associated to asymptomatic plants under normal growth conditions [39].

This is the first described virus sequence and EPRV inserted in Maqui berry Genome. Until now, the only described similarities between these hosts plants, *P. hybrida* and *A. chilensis*, were their characteristic dark purple colors, directly associated to anthocyanin production [5,43], and the fact that both are endemic from South America, Argentina and Chile, respectively [8,38]. Additionally, this work showed the importance and application of next generation sequencing and bioinformatics in the identification and characterization of new viral sequences inserted in plants genomes.

## 4. Materials and Methods

### 4.1. DNA Sequencing and Data Processing

The *Aristotelia chilensis virus 1* (AcV1) (GenBank accession number KJ094314) was identified from the recently sequenced genome of Maqui (*Aristotelia chilensis*) sample M152, plant #6 [44], which was collected at the 10th region of Chile. Total Maqui DNA was extracted from young leaves using a modified grapes method [45]. A total of 5 µg of DNA were used to prepare an Illumina paired-end (IPE) shotgun library using the Nextera kit (Illumina, San Diego, California, USA). This library was sequenced using the MiSeq Illumina platform (220 bp paired-end reads). The *de novo* assembly of the draft genome was performed using the MIRA assembler tool [46]. Viral sequences were identified using BLASTx, comparing the obtained nucleotides sequences against the non-redundant (NR) protein database from NCBI, followed by a taxonomic classification using MEGAN software [47]. 

### 4.2. Sequence Analysis and Genome Comparison

For the annotation process, all viral contigs were used as input for the Artemis tool [11]. All identified ORFs were annotated using BLASTp tool against the NR protein database of NCBI. Sequence comparisons against plant pararetroviruses of *Caulimoviridae* family and Ty3 retrotransposon were performed using multiple alignments tool available in CLUSTAL OMEGA [18]. The visualization was performed using the multiple alignment sequences editor Jalview [48]. The representative viruses of *Caulimoviridae* family that we used were: CaMV, PVCV, CsVMV, DMV, DMV-10-DR, DMV-10-NZ, CERV, FMV, CitPRV *Strawberry vein banding virus* (SVBV), *Blueberry red ringspot virus* (BRRV), *Peanut chlorotic streak virus* (PCISV), *Soybean chlorotic mottle virus* (SoyCMV), *Commelia yellow mottle virus* (CoyMV), RTBV, *Sugarcane bacilliform virus* (SCBV), CSSV and the retrotransposon Ty3 from *S. cerevisiae.*

The VISTA [35] comparison tool and MLAGAN align [34] were used to compare AcV1 against virus with similar phylogenetic characteristics. The alignment was built using a conservation threshold of 70% as identity, over a window of 100 bp between viral genomes in comparison to AcV1.

A phylogenetic tree was built with the multiple alignment of RT under bootstrap analysis (1000 replicates) [30], using Molecular Evolutionary Genetics Analysis (MEGA) software [28]. For the evolutionary comparisons, we used the RT region of the *Caulimoviridae* family and Ty3 retrotransposon. This approach was constructed using the Maximum Likelihood method [28]. The length of the branches is proportional to the evolutionary distances computed using the Dayhoff matrix based approach [29]. 

### 4.3. RNA Sequencing and Data Processing

Total RNA from Maqui berry samples was isolated using TRIzol^®^ Reagent (Invitrogen, San Diego, California, Country). RNA quality was determined by the quotient of the 28S to 18S ribosomal RNA electropherogram peak using a Bioanalyzer Chip RNA 7500 series II (Agilent, Santa Clara, California, USA). Four libraries were prepared using the TruSeq RNA Sample Preparation Kit v2 (Illumina) according to the manufacturer’s instructions. First, magnetic beads containing poly-T molecules were used to isolate poly(A) mRNA from 1 μg of total RNA. Then, samples were fragmented by fragmentation buffer and reverse transcribed into cDNA with a PrimeScript™ 1st Strand cDNA Synthesis Kit (TaKaRa, Dalian, China). Next, end repair and A-base tailing was purified using the QIAquick PCR Purification Kit (QIAGEN, Hilden, Germany). Finally, Illumina adapters were ligated to the cDNA fragments. Each library had an insert size of 300 bp sequenced in paired-end reads of 150 bp using a MiSeq Desktop Sequencer.

Raw reads were used to identify expression levels of all three genes associated to AcV1. In order to align raw reads against our virus genome, Bowtie2 [49] was employed, followed by the software IGV (Integrative Genomics Viewer) [50]. This allowed the identification of reads distribution along the viral whole genome sequence and to obtain a general overview of AcV1 genomic structure.

## Supplementary Files

Supplementary File 1

## References

[1] Hansen C., Heslop-Harrison J. (2004). Sequences and phylogenies of plant pararetroviruses, viruses, and transposable elements. Adv. Bot. Res..

[2] Barba M., Czosnek H., Hadidi A. (2014). Historical perspective, development and applications of next-generation sequencing in plant virology. Viruses.

[3] Prabha K., Baranwal V.K., Jain R.K. (2013). Applications of next generation high throughput sequencing technologies in characterization, discovery and molecular interaction of plant viruses. Indian J. Virol..

[4] Araya H., Clavijo C., Herrera C. (2006). Capacidad antioxidante de frutas y verduras cultivados en Chile. Arch. Latinoam. Nutr..

[5] Escribano-bailón M.T., Alcalde-eon C., Muñoz O., Rivas-gonzalo J.C., Santos-buelga C. (2006). Anthocyanins in Berries of Maqui [Aristotelia chilensis (Mol.) Stuntz]. Phytochem. Anal..

[6] Schreckinger M., Wang J. (2010). Antioxidant capacity and *in vitro* inhibition of adipogenesis and inflammation by phenolic extracts of Vaccinium floribundum and Aristotelia chilensis. J. Agric. Food Chem..

[7] Céspedes C.L., El-Hafidi M., Pavon N., Alarcon J. (2008). Antioxidant and cardioprotective activities of phenolic extracts from fruits of Chilean blackberry Aristotelia chilensis (Elaeocarpaceae), Maqui. Food Chem..

[8] Miranda-Rottmann S., Aspillaga A.A., Pérez D.D., Vasquez L., Martinez A.L.F., Leighton F. (2002). Juice and phenolic fractions of the berry Aristotelia chilensis inhibit LDL oxidation *in vitro* and protect human endothelial cells against oxidative stress. J. Agric. Food Chem..

[9] Harper G., Hull R., Lockhart B., Olszewski N. (2002). Viral sequences integrated into plant genomes. Annu. Rev. Phytopathol..

[10] Pfeiffer P., Hohn T. (1983). Involvement of reverse transcription in the replication of cauliflower mosaic virus: A detailed model and test of some aspects. Cell.

[11] Rutherford K., Parkhill J., Crook J. (2000). Artemis: Sequence visualization and annotation. Bioinformatics (Oxf. Engl.).

[12] Hull R., Sadler J., Longstaff M. (1986). The sequence of carnation etched ring virus DNA: Comparison with cauliflower mosaic virus and retroviruses. EMBO J..

[13] Hagen L., Jacquemond M., Lepingle A., Lot H., Tepfer M. (1993). Nucleotide sequence and genomic organization of cacao swollen shoot virus. Virology.

[14] Hay J.M., Jones M.C., Blakebrough M.L., Dasgupta I., Davies J.W., Hull R. (1991). An analysis of the sequence of an infectious clone of rice tungro bacilliform virus, a plant pararetrovirus. Nucleic Acids Res..

[15] Richert-Pöggeler K.R., Shepherd R.J. (1997). Petunia vein-clearing virus: A plant pararetrovirus with the core sequences for an integrase function. Virology.

[16] Roy A., Shao J., Schneider W.L., Hartung J.S., Brlansky R.H. (2014). Population of endogenous pararetrovirus genomes in carrizo citrange. Genome Announc..

[17] Richins R. (1987). Sequence of figwort mosaic virus DNA (caulimovirus group). Nucleic Acids Res..

[18] Sievers F., Wilm A., Dineen D. (2011). Fast, scalable generation of high-quality protein multiple sequence alignments using Clustal Omega. Mol. Syst..

[19] Koonin E.V., Mushegian A.R., Ryabov E.V., Dolja V.V. (1991). Diverse groups of plant RNA and DNA viruses share related movement proteins that may possess chaperone-like activity. J. Gen. Virol..

[20] Kasteel D.T., Perbal M.C., Boyer J.C., Wellink J., Goldbach R.W., Maule A.J., van Lent J.W. (1996). The movement proteins of cowpea mosaic virus and cauliflower mosaic virus induce tubular structures in plant and insect cells. J. Gen. Virol..

[21] Piqué M., Mougeot J.L., Geldreich A., Guidasci T., Mesnard J.M., Lebeurier G., Yot P. (1995). Sequence of a cauliflower mosaic virus strain infecting solanaceous plants. Gene.

[22] Xiong Y., Eickbush T. (1990). Origin and evolution of retroelements based upon their reverse transcriptase sequences. EMBO J..

[23] Hansen L., Sandmeyer S. (1990). Characterization of a transpositionally active Ty3 element and identification of the Ty3 integrase protein. J. Virol..

[24] Malik H., Eickbush T. (2001). Phylogenetic analysis of ribonuclease H domains suggests a late, chimeric origin of LTR retrotransposable elements and retroviruses. Genome Res..

[25] Kano H., Koizumi M., Noda H., Hibino H. (1992). Nucleotide sequence of capsid protein gene of rice tungro bacilliform virus. Arch. Virol..

[26] Almeyda C.V., Eid S.G., Saar D., Samuitiene M., Pappu H.R. (2014). Comparative analysis of endogenous plant pararetroviruses in cultivated and wild Dahlia spp.. Virus Genes.

[27] Geering A.D.W., Maumus F., Copetti D., Choisne N., Zwickl D.J., Zytnicki M., McTaggart A.R., Scalabrin S., Vezzulli S., Wing R.A. (2014). Endogenous florendoviruses are major components of plant genomes and hallmarks of virus evolution. Nat. Commun..

[28] Tamura K., Peterson D., Peterson N., Stecher G., Nei M., Kumar S. (2011). MEGA5: Molecular evolutionary genetics analysis using maximum likelihood, evolutionary distance, and maximum parsimony methods. Mol. Biol. Evol..

[29] Dayhoff M., Schwartz R. (1978). A model of evolutionary change in proteins. Atlas Protein Seq. Struct..

[30] Felsenstein J. (1985). Confidence limits on phylogenies: An approach using the bootstrap. Evolution.

[31] Calvert L.A., Ospina M.D., Shepherd R.J. (1995). Characterization of cassava vein mosaic virus: A distinct plant pararetrovirus. J. Gen..

[32] Medberry S. (1990). Properties of Commelina yellow mottle virus’s complete DNA sequence, genomic discontinuities and transcript suggest that it is a pararetrovirus. Nucleic Acids.

[33] He X., Fütterer J., Hohn T. (2002). Contribution of downstream promoter elements to transcriptional regulation of the rice tungro bacilliform virus promoter. Nucleic Acids Res..

[34] Brudno M., Do C.B., Cooper G.M., Kim M.F., Davydov E., Green E.D., Sidow A., Batzoglou S. (2003). LAGAN and Multi-LAGAN: Efficient tools for large-scale multiple alignment of genomic DNA. Genome Res..

[35] Frazer K.A., Pachter L., Poliakov A., Rubin E.M., Dubchak I. (2004). VISTA: Computational tools for comparative genomics. Nucleic Acids Res..

[36] Gayral P., Noa-Carrazana J.C., Lescot M., Lheureux F., Lockhart B.E.L., Matsumoto T., Piffanelli P., Iskra-Caruana M.L. (2008). A single Banana streak virus integration event in the banana genome as the origin of infectious endogenous pararetrovirus. J. Virol..

[37] Lockhart B.E., Menke J., Dahal G., Olszewski N.E. (2000). Characterization and genomic analysis of tobacco vein clearing virus, a plant pararetrovirus that is transmitted vertically and related to sequences integrated in the host genome. J. Gen. Virol..

[38] Richert-Pöggeler K.R., Noreen F., Schwarzacher T., Harper G., Hohn T. (2003). Induction of infectious petunia vein clearing (pararetro) virus from endogenous provirus in petunia. EMBO J..

[39] Noreen F., Akbergenov R., Hohn T., Richert-Pöggeler K.R. (2007). Distinct expression of endogenous Petunia vein clearing virus and the DNA transposon dTph1 in two Petunia hybrida lines is correlated with differences in histone modification and siRNA production. Plant J. Cell Mol. Biol..

[40] Chabannes M., Iskra-Caruana M.L. (2013). Endogenous pararetroviruses-a reservoir of virus infection in plants. Curr. Opin. Virol..

[41] Staginnus C., Richert-Pöggeler K.R. (2006). Endogenous pararetroviruses: Two-faced travelers in the plant genome. Trends Plant Sci..

[42] Staginnus C., Iskra-Caruana M.L., Lockhart B., Hohn T., Richert-Pöggeler K.R. (2009). Suggestions for a nomenclature of endogenous pararetroviral sequences in plants. Arch. Virol..

[43] Beld M., Martin C., Huits H., Stuitje A.R., Gerats A.G. (1989). Flavonoid synthesis in Petunia hybrida: Partial characterization of dihydroflavonol-4-reductase genes. Plant Mol. Biol..

[44] Villacreses J., Rojas-Herrera M., Sanchez C., Blanc N., Espinoza L., Pastor G., Hewstone N., Undurraga S.F., Alzate J.F., Manque P., Maracaja-Coutinho V., Polanco V. Draft genome sequence of the antioxidant-rich plant Maqui Berry (*Aristotelia chilensis*).

[45] Lodhi M., Ye G., Weeden N., Reisch B. (1994). A simple and efficient method for DNA extraction from grapevine cultivars and Vitis species. Plant Mol. Biol..

[46] Chevreux B., Pfisterer T., Drescher B. (2004). Using the miraEST assembler for reliable and automated mRNA transcript assembly and SNP detection in sequenced ESTs. Genome.

[47] Huson D.H., Mitra S., Ruscheweyh H.J., Weber N., Schuster S.C. (2011). Integrative analysis of environmental sequences using MEGAN4. Genome Res..

[48] Waterhouse A.M., Procter J.B., Martin D.M.A., Clamp M., Barton G.J. (2009). Jalview Version 2 a multiple sequence alignment editor and analysis workbench. Bioinformatics (Oxf. Engl.).

[49] Langmead B., Salzberg S.L. (2012). Fast gapped-read alignment with Bowtie 2. Nat. Meth..

[50] Thorvaldsdóttir H., Robinson J.T., Mesirov J.P. (2013). Integrative Genomics Viewer (IGV): High-performance genomics data visualization and exploration. Brief. Bioinform..

